# Impact of organic contaminants in soils from Important Bird and Biodiversity areas

**DOI:** 10.1007/s11356-024-35274-7

**Published:** 2024-10-22

**Authors:** Maria Dulsat-Masvidal, Carlos Ciudad, Octavio Infante, Rafael Mateo, Silvia Lacorte

**Affiliations:** 1 Department of Environmental Chemistry, IDAEA-CSIC, Jordi Girona 18-26, 08034 Barcelona, Spain; 2 SEO/BirdLife, Melquiades Biencinto, 34, 28053 Madrid, Spain; 3 Instituto de Investigación en Recursos Cinegéticos (IREC), CSIC-UCLM-JCCM, 13005 Ciudad Real, Spain

**Keywords:** Important Bird and Biodiversity Areas, Organic contaminants, Soils

## Abstract

Soils act as sinks for many organic contaminants, posing a threat to biodiversity and essential ecosystem services. In this study, we assessed the contamination status of soils in 140 Important Bird and Biodiversity Areas (IBAs) in Spain. Fifty-two organic contaminants including organochlorine pesticides (OCPs), organophosphorus pesticides (OPPs), polychlorinated biphenyls (PCBs), polycyclic aromatic hydrocarbons (PAHs), and plasticizers or plastic related such as phthalates, bisphenol A, nonylphenol, and organophosphate esters (OPEs) were analyzed by gas chromatography coupled to tandem mass spectrometry (GC–MS/MS). The mean soil concentration ranged from 1.41 to 917 ng/g and plasticizer and PAHs were detected at the highest concentrations, while OCPs were the most frequently detected. Hierarchical clustering on principal components (HCPC) and land use data associated PCBs with artificial land, phthalates with industrial sites and incineration plants and PAHs with burned areas, and in a lesser extent pesticides with agricultural activities. A tier I environmental risk assessment (ERA) was performed to identify the most impacted natural areas and the most concerning compounds. Out of the 140 IBAs, 95 presented at least one compound at high-risk concentrations (RQ > 1) for soil organisms. The OPPs chlorpyrifos and malathion, together with the PAH benzo[b]fluoranthene, were detected at high-risk concentrations. Overall, this study highlights the widespread presence of organic contaminants in areas of high natural value and the importance of implementing monitoring studies to identify potential contaminated sites that require conservation and remediation actions for the protection of biodiversity.

**Supplementary Information:**

The online version contains supplementary material available at 10.1007/s11356-024-35274-7.

## Introduction

Soils are non-renewable resources and represent a compartment of global biodiversity, which is crucial for providing ecosystem services such as agricultural and biomass production, supply of raw materials, filtration of contaminants, regulation of water, and nutrient cycling (Ferreira et al. 2022). However, in recent decades, there has been an increasing concern regarding the rapid degradation of soils, which have the potential to negatively impact landscapes and ecosystems. Soils are globally threatened mostly by human activities, such as unsustainable practices in agriculture and forestry, industry emissions, waste discharges, and soil sealing through urbanization and infrastructures (European Commission 2020). Soil contamination, either from natural or anthropogenic sources, can represent a serious impact on biodiversity and ecosystem functions (Liu et al. 2023). In the EU, most soils are considered unhealthy as 2.8 million sites are known to be contaminated (European Commission 2020). Recently, the EU proposed a new soil monitoring law to protect and restore soils and to ensure their sustainable use. This new proposal also involves the identification, investigation, and assessment of contaminated sites (European Commission 2023a).

Soils are sinks of legacy contaminants such as organochlorine pesticides (OCPs), widely used in the past in domestic and agricultural applications, and polychlorinated biphenyls (PCBs) used for industrial purposes. Soil contamination can be local, where there is a clear source of contaminants affecting a limited area, or diffuse, which is much harder to manage as there is not a directly apparent source of pollution and it can affect a very large area (FAO 2015). Despite important efforts have been made to restrict, ban, and eliminate the production and use of these compounds through international treaties (Stockholm Convention 2019), residues are present in the global environment, even in remote areas with low human footprint due to their persistency and long-range atmospheric transport (Kim et al. 2021; Mishra et al. 2022). After the ban on OCPs, organophosphorus pesticides (OPPs) such as chlorpyrifos, malathion, and chlorfenvinphos have been extensively used in agriculture, and although they are more easily degraded, they trigger toxicological adverse effects. Other important soil contaminants are polycyclic aromatic hydrocarbons (PAHs), which are formed by the combustion of organic matter such as natural sources through wildfires, volcanic activity, or geological formation of fossil fuels, or from anthropogenic sources such as industrial emissions, traffic, and heating systems for households (gas, wood, or coal burning) (Rengarajan et al. 2015). They impact soils as they have mutagenic and carcinogenic properties (Shukla et al. 2022). Finally, high-volume production chemicals such as phthalates, organophosphate esters (OPEs), bisphenol A, and nonylphenol are ubiquitous in the environment (Wang et al. 2020) and have raised concern due to their capacity to bioaccumulate and biomagnify along the trophic chains and affect humans and wildlife (Greaves and Letcher 2017; Zhang et al. 2022a, b).

The contamination of soils has been studied mostly in urban and agricultural areas and there are few monitoring studies on soils from natural areas (Aichner et al. 2013). Filling this gap of knowledge is of utmost importance, as natural areas are crucial for the conservation of global biodiversity and can play an important role in the retention of contaminants. Furthermore, in the actual context of climate change, the re-emission of persistent compounds from soil is more likely to occur, and in fact, this phenomenon has been already reported in European soils (Degrendele et al. 2016; Ren et al. 2019). To assess the health of soils, many countries have implemented long-term surveys focused on metals and organic matter, but lack of harmonized soil monitoring system for organic contaminants, and the real extent of soil contamination, especially for emerging contaminants, is still unknown (FAO 2015).

Important Bird and Biodiversity Areas (IBAs) are sites designated by the non-profit organization BirdLife International to preserve natural areas with high value for birds and biodiversity conservation (Donald et al. 2019). In recent studies, freshwaters from IBAs have been found to be impacted by organic micropollutants, evidencing the underlying pressure of contaminants in these natural areas (Dulsat-Masvidal et al. 2023). In the present study, we performed a monitoring survey to determine 52 organic contaminants including legacy (PAHs, OCPs, PCBs) and emerging (OPEs, OPPs, phthalate esters (PAEs), bisphenol A (BPA), nonylphenol (NP)) in soils from 140 IBAs in Spain with the aim to assess the pollution patterns in these natural areas and identify the distribution and potential sources of the studied contaminants and the risk they may pose to soil conservation.

## Materials and methods

### Chemicals and reagents

A total of 52 organic contaminants were investigated in the present study, including 16 PAHs, 14 OCPs, 3 OPPs, 7 marker PCBs congeners, 4 OPEs, 6 PAEs, BPA, and technical NP. Surrogate standards consisted of deuterated PAHs solution mix containing naphthalene d-8, acenaphthene d-10, phenanthrene d-10, chrysene d-12, and perylene d-12, purchased from Sigma-Aldrich (Darmstadt, Germany and St. Louis, MO, USA). These deuterated PAHs elute along the chromatogram and were used to quantify compounds present in each retention time window. Further details of the analyte standards are provided in Table S1. Calibration standards were prepared in hexane. Solvents used were hexane from Merck (Darmstadt, Germany) and dichloromethane from Carlo Erba Reagents (Sabadell, Spain).

### Sample collection

Soil samples were collected in Spain between 2019 and 2020 from 140 IBAs representative of 7 ecosystems: agricultural, Atlantic forest, Mediterranean forest, riparian forest, rocky mountain, inland aquatic, and coastal habitats (Fig. S1, Table S2). Soil was sampled using a scoop and avoiding the first 4–5 cm upper soil, as the top surface is affected by sunlight, erosion, and deterioration which could produce the degradation of some contaminants. Each sample consisted of 0.8 and 1 kg of soil composed of a minimum of eight subsamples collected 10 m apart to enhance representativeness. We georeferenced the sampling points and provided an observational land description (e.g. presence of trash). Samples were placed in glass containers and sent refrigerated to the laboratory, where they were dried at 40 °C until constant weight using a natural convection oven (J.P. Selecta, Abrera, Barcelona). Soils were then sieved through 500- and 125-µm stainless steel mesh and the latter fraction was analyzed. Samples were preserved at 4 °C in amber glass vials until chemical analysis.

### Sample treatment and chemical analysis

One gram of sample was weighed in 60-mL glass centrifuge tubes and spiked with 50 ng of the internal standard solution. As extraction solvent, 30 mL of hexane:dichloromethane (1:1) were added, following a previous procedure for the analysis of contaminants in dust (Velázquez-Gómez et al. 2018). Samples were vortexed (1 min) and ultra-sonicated (10 min), and this procedure was repeated three times without changing the solvent. Finally the samples were centrifuged (10 min, 1560 rcf, 20 °C). The supernatant was collected and transferred to a 40-mL amber vial and concentrated to 2 mL using a gentle N_2_ flow at 20 °C using a TurboVap® LV (Caliper Lifesciences, Uppsala, Sweden). Clean-up was performed with 5-g Bond Elut Florisil cartridges (Agilent Technologies, Santa Clara, CA, USA). Cartridges were conditioned with 30 mL hexane: dichloromethane (1:1) dropwise, and samples were loaded and eluted with 30 mL of the same solvent mix. The extract was evaporated again using the TurboVap® LV to near dryness and transferred to chromatographic vials to a final volume of 0.5 mL in hexane. If some solid particles appeared during the final preconcentration step, extracts were filtered through 0.2 µm × 13 mm nylon filters (Clarify, Phenomenex, Torrance, USA).

Samples were analyzed by gas chromatography coupled to a triple quadrupole (QqQ) mass spectrometer (Agilent 7890A chromatograph and 7000A MS from Agilent Technologies, Santa Clara, CA, USA) with electron ionization (EI) at 70 eV. Separation of target compounds was achieved using a HP-5MS Agilent column (30 m × 0.25 mm internal diameter, 0.25 µm film thickness). The initial temperature was set at 70 °C and kept for 1 min, then increased to 175 °C in 4 min, from 175 to 235 °C in 20 min, and to 305 °C in 8 min. The slow gradient elution that lasted 60 min allowed the multiresidue analysis to resolve the 52 contaminants belonging to the different chemical families. Compounds were identified by retention time and by the specific MS/MS transition (Velázquez-Gómez et al. 2018), so that all of them could be determined. The injection sequence included the analysis of a standard at 0.05 ng/µL every 15 samples that was used as injection standard. If the response varied within 30% of the theoretical (initial) response, a cleaning procedure of the column, change of liner, or ultimately cleaning of the ion source was undertaken. The data was processed by Mass Hunter Quantitative software. Internal standard quantification was performed and Table S3 indicates the compounds eluting in each time window and the deuterated PAH standards used for quantification. Concentrations are given as ng/g dry weight (dw).

### Quality control/quality analysis

The analytical method was assessed for precision, accuracy, linearity, sensitivity, selectivity, and extraction efficiency. Inter-day precision was determined by injecting a 0.05 ng/µL standard in five different days. Linearity was studied over a concentration range of 0.001–0.8 ng/µL. To evaluate the extraction efficiency (recoveries), a pristine and acetone washed freeze-dried soil was spiked with 50 ng/g of a mixture of 52 contaminants (*n* = 5) and analyzed by the described analytical procedure. Unspiked soil was also extracted to guarantee the absence of initial contaminant contribution. Instrumental limits of detection (IDL) were calculated as the amount of analyte that gives a signal-to-noise ratio of 3 (S/N = 3) using the 0.001 ng/µL standard. Method detection limits (MDL) were calculated as the concentration that give a S/N = 3 using the 50 ng/g spiked soil, except for phthalates and OPEs that had a strong blank contribution and then the MDL were calculated as three times the standard deviation of the blank contribution (*n* = 10). Quality parameters including recoveries and MDL of the target compounds are indicated in Table S3. All target analytes were recovered within the 70–130%, except for PCB118 that had a recovery of 135%, and dibenzo[a,h]anthracene, benzo[ghi]perylene, DMP, DBP, BBZP, TDCPP, and EHDPHP were recovered in less than 50%. The method showed good precision and the MDL were within the 0.02 to 13.8 ng/g dw except for phthalates and OPEs that much higher levels were obtained due to the blank contribution. Samples’ values below MDL were given a value of zero to avoid overestimation in statistical analysis.

### Data analysis

To assess the geographical distribution of the detected compounds, we considered spatial data around each sampling point. We estimated a dominant influence of contaminants sources within a 10-km buffer radius, based on previous contaminant deposition studies (Hu et al. 2021; Rossini et al. 2005). We collected spatial information including Corine land use grouped in major classes (agricultural, artificial, and natural surface), number of industrial sites, incineration plants, roads density considering the total length of roads in a 10-km area, and surface burned areas in the period of 5 years before sampling. Spatial information was expressed as percentage. For each sampling point, the total organic carbon (TOC) was obtained from the topsoil soil organic carbon (LUCAS) (de Brogniez et al. 2015). The spatial information used here is detailed in Table S4.

The spatial data was obtained from the following databases: the Corine Land Cover 2018 (https://land.copernicus.eu/), Spanish Ministry for Ecological Transition and the Demographic Challenge services (https://www.miteco.gob.es), European Forest Fire Information System (https://effis.jrc.ec.europa.eu/), and European Soil Data Centre (https://esdac.jrc.ec.europa.eu/).

To characterize the contamination profile of each IBA, we performed a hierarchical clustering on principal component (HCPC) analysis with log x + 1 concentrations of compounds detected in more than 10% of soils and considering the spatial information within 10 km of each sampling point. The Kaiser–Meyer–Olkin (KMO) was used to assess the suitability of the principal component analysis (PCA), where KMO > 0.5 indicates variables enough interdependent for PCA (Dziuban et al. 1979).

Spatial analysis and maps were performed in open-source software QGIS (version 3.18.2). Statistical analyses were performed in R studio (R version 4.0.3). FactoMineR package was used for HCPC analysis and figures were elaborated using ggplot2 package.

### Environmental risk assessment

A tier I environmental risk assessment (ERA) was performed for the detected compounds in soils. Risk quotients (RQs) were assessed as the ratio between the measured environmental concentrations (MEC) for each compound *(i)* and the predicted no-effect concentration (PNEC) (Eq. 1).

1 $${RQ}_{i}=\frac{{MEC}_{i}}{{PNEC}_{i}}$$

There is limited ecotoxicological data in soil organisms, compared to freshwater organisms, and obtaining the toxicity data is not straightforward. To perform a unified estimation of RQs for all compounds, all values were extrapolated using the equilibrium partitioning method following the European Technical Guidance on Risk Assessment (European Commission 2003) considering the organic carbon–water partition coefficient (K_oc_) for each compound obtained from EPI Suite (EPA 2012), the weight fraction of organic carbon in soil (f_oc_), and the bulk density of wet suspended matter (RHO_susp_) obtained from the European Technical Guidance on Risk Assessment (European Commission 2003) (Eq. 2). The lowest PNEC values in freshwater were obtained from NORMAN database (NORMAN 2022). The calculation and full list of PNEC values for soil is detailed in Table S5.

2 $${PNEC}_{comp.}=\frac{{K}_{oc}. {f}_{oc}}{{RHO}_{susp}}\cdot {PNEC}_{water}\cdot 1000$$

The prioritization of the most concerning compounds among IBAs was performed calculating the RQ_f,i_ described by Zhou et al. (2019), where *f* corresponds to the frequency of MECs exceeding PNEC (RQ > 1) (Eq. 3):

3 $${RQ}_{f, i}={\sum }_{i=1}^{n}\frac{{MEC}_{i}}{{PNEC}_{i}}\times \frac{Number\ of\ samples\ where\ {MEC}_{i}>{PNEC}_{i}}{Total\ number\ of\ IBAs\ (n=140)}$$

## Results and discussion

### Levels and occurrence of contaminants in soils

**Fig. 1:**
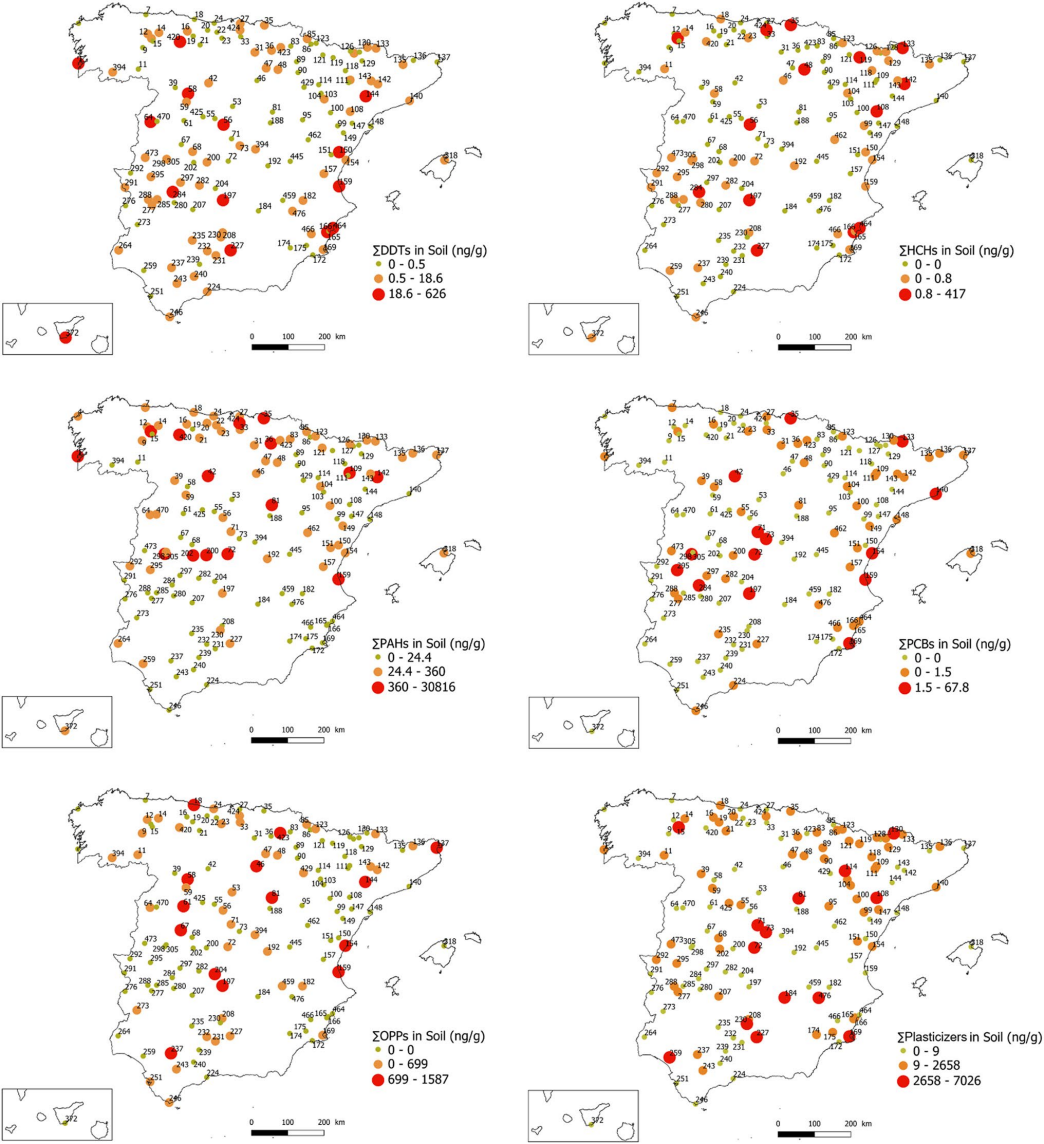
Spatial distribution of the most relevant chemical families detected in IBAs soils, classified in percentiles: minimum to 50%, 50 to 90%, and 90% to maximum for each chemical group. Numbers in the maps indicate IBA codes (Table S2 for IBA identification)

Fifty target compounds out of 52 analyzed were detected in soils from Spanish IBAs (Table 1). According to the detection frequency, the chemical families detected followed the order: OCPs > PAHs > plasticizers > PCBs > OPPs > OPEs. The mean concentration of target compounds followed the order: plasticizers > PAHs > OPPs > OCPs > OPEs > PCBs. Figure 1 maps the distribution of the most relevant chemical families classified in percentiles according to their concentrations. The site-specific contaminant concentrations are available in Table S2 from supplementary material.

**Table 1:** Detected target compounds in soils (*n* = 140) ordered by detection frequency within each chemical family, number of occurrences (*N*) and detection frequency (%), mean ± S.E. (standard error), minimum and maximum concentrations, expressed in ng/g dw. Endosulfan not detected. The values in bold indicate the total sums of the chemical families

Compound	*N* (%)	Mean ± S.E	Min	Max
4,4′-DDE	107 (76)	8.6 ± 3.22	0.07	358
4,4′-DDD	39 (28)	1.56 ± 0.57	0.06	56.2
4,4′-DDT	33 (24)	9.28 ± 3.28	2.67	308
2,4′-DDD	18 (13)	0.12 ± 0.04	0.13	2.39
2,4′-DDT	12 (9)	0.62 ± 0.26	1.24	27.7
2,4′-DDE	12 (9)	0.09 ± 0.03	0.09	3.01
β-HCH	40 (29)	0.09 ± 0.03	0.02	2.46
γ-HCH	27 (19)	3.44 ± 2.96	0.02	410
α-HCH	8 (6)	1.02 ± 0.68	2.43	90.7
ɗ-HCH	1 (0.7)	-	0.51	0.51
HCB	18 (13)	0.28 ± 0.18	0.07	24.2
HCBD	7 (5)	0.32 ± 0.23	0.27	31.1
**∑OCPs**	**122 (87)**	**25.4 ± 7.65**	**0.03**	**626**
Pyrene (HMW)	85 (61)	37.4 ± 19	0.81	2427
Benzo[b]fluoranthene (HMW)	75 (54)	60.3 ± 46.1	1.04	6462
Benzo[k]fluoranthene (HMW)	74 (53)	53.1 ± 40.6	1.52	5687
Benzo[ghi]perylene (HMW)	63 (45)	23.9 ± 13.7	2.57	1909
Fluoranthene (HMW)	59 (42)	40.9 ± 25.5	4.17	3561
Benzo[a]anthracene (HMW)	56 (40)	23.1 ± 15.4	2.82	2153
Phenanthrene (LMW)	48 (34)	8.75 ± 4.78	1.25	663
Indeno[1,2,3-cd] pyrene (HMW)	38 (27)	31.3 ± 20.7	7.16	2889
Chrysene (HMW)	37 (26)	12.0 ± 6.6	3.92	917
Acenaphthene (LMW)	37 (26)	3.33 ± 0.6	4.92	52.4
Benzo[a]pyrene (HMW)	25 (18)	31.4 ± 21.7	13.85	3027
Anthracene (LMW)	19 (14)	6.8 ± 5.5	0.62	766
Fluorene (LMW)	16 (11)	1.16 ± 0.56	0.69	57.2
Naphthalene (LMW)	9 (6)	5.13 ± 2.92	1.4	386
Dibenzo[a,h]anthracene (HMW)	9 (6)	3.34 ± 2.13	6.19	259
Acenaphthylene (LMW)	7 (5)	0.33 ± 0.14	1.45	14.1
**∑PAHs**	**96 (69)**	**342 ± 220**	**1.4**	**30,816**
NP	29 (21)	1155 ± 100	1247	3488
DEP	16 (11)	181 ± 56.7	534	5475
DiBP	16 (11)	100 ± 35.8	175.8	2921
DEHP	11 (8)	149 ± 49.2	1223	4073
DMP	10 (7)	0.86 ± 0.32	5.5	32.3
BPA	7 (5)	3.58 ± 1.47	37.1	148
DBP	6 (4)	19.2 ± 8.42	200	771
BBZP	3 (2)	0.76 ± 0.44	25.1	41.4
**∑Plasticizers**	**70 (50)**	**917 ± 118**	**5.5**	**7026**
PCB 153	54 (39)	0.46 ± 0.15	0.08	18.4
PCB 101	30 (21)	0.06 ± 0.02	0.03	1.86
PCB 138	28 (20)	0.43 ± 0.19	0.07	24.7
PCB 180	25 (18)	0.44 ± 0.18	0.11	22.7
PCB 52	12 (9)	0.01 ± 0	0.02	0.38
PCB 28	2 (0.7)	0.01 ± 0	0.42	0.5
PCB 118	2 (0.7)	-	0.1	0.15
**∑PCBs**	**61 (44)**	**1.41 ± 0.54**	**0.02**	**67.8**
Chlorpyrifos	38 (27)	162 ± 27.7	125	1587
Malathion	17 (12)	0.9 ± 0.38	1.03	48.3
Chlorfenvinphos	1 (0.7)	-	79.3	79.3
**∑OPPs**	**51 (36)**	**163 ± 27.7**	**1.03**	**1587**
TBOEP	4 (3)	7.03 ± 5.91	42.6	824
EHDPHP	2 (0.7)	2.3 ± 1.68	114	207
TDCPP	1 (0.7)	-	27.6	27.6
TDCEP	1 (0.7)	-	5.84	5.84
**∑OPEs**	**7 (5)**	**9.56 ± 6.32**	**5.84**	**851**

∑OCPs were the most frequently detected compounds in soils, found in 87% of the IBAs at concentrations ranging from 0.03 to 626 ng/g (Table 1). ∑DDTs were detected in 79% of the samples and contributed to 80% of ∑OCPs. Despite the high ubiquity, the 50th percentile of ∑DDTs was 0.5 ng/g indicating that most samples presented low concentrations (Fig. 1). The highest levels of ∑DDTs were found at IBAs 064 (Rio Huebra–Arribes del Duero, Salamanca), 144 (Cogul–Alfes, Lleida), and 001 (Islas Cies, Pontevedra) (Fig. 1) that had concentrations from 483 to 626 ng/g dw. Among DDTs isomers, the metabolite 4,4′-DDE was detected in 76% of the samples at levels from 0.07 to 358 ng/g, followed by 4,4′-DDD present in 28% at concentrations from 0.06 to 56.2 ng/g, and 4,4′-DDT detected in 24% of the samples at levels from 2.67 to 308 ng/g. In the environment, DDT is aerobically degraded to DDE or anaerobically degraded to DDD (Qu et al. 2019). Only six samples (IBAs 197, 424, 36, 200, 73, 59; see Figure S1 and Table S1 2 for details of those IBAs) showed higher concentrations of DDD than DDE, indicating a higher prevalence of anaerobic degradation of DDT. Despite being a long-banned pesticide, DDTs and its metabolites are still one of the most widespread insecticide residues in European agricultural soils, and 4,4′-DDE is the most prevalent compound (Silva et al. 2019). ∑HCHs were present at 39% of the samples, but their concentrations were generally found at trace levels. The 90th percentile concentration for ∑HCHs was 0.8 ng/g, indicating that nearly all samples had concentrations below this level and indicated a low-level historical pollution in IBAs from Spain (Fig. 1). Technical HCHs, consisted in 90% of γ-HCH isomer, also known as lindane, are used as insecticide. Although β-HCH was detected in 29% of the samples at concentrations ranging from 0.02 to 2.46 ng/g, γ-HCH was present in 19% of the samples but at much higher concentrations (from 0.02 to 410 ng/g), and α-HCH was present only in 6% of the samples at levels from 2.43 to 90.7 ng/g. IBA 119 (Oturia–Cancias, Huesca, north east Spain, Fig. 1) was the most impacted by HCHs as with ∑HCHs of 417 ng/g following the pattern: γ-HCH at 410 ng/g, α-HCH at 4.58 ng/g, β-HCH at 2.34 ng/g, and δ-HCH at 0.51 ng/g. IBA 119 is located in Sardas and Bailín-Sabiñánigo (Aragón, Spain), a landfill used by the Inquinosa company which produced 160000 tons of HCHs waste between 1975 and 1988 (Gómez-Lavín et al. 2018). Our results indicate that the IBA close to this area is still strongly affected by the historic production of lindane. HCBD was seldom detected among soils samples.

∑PAHs were detected in 69% of the IBAs at concentrations from 1.4 to 30816 ng/g. As it is shown in Fig. 1, the 50th percentile was of 24.4 ng/g, indicating that half of the sampling locations presented concentrations below this value. This median value is higher than the other chemical groups, meaning that overall, PAHs were present at relatively high concentrations (Fig. 1). Northern Spanish IBAs were more impacted than southern IBAs, with concentrations within the 75th (369 ng/g) and 90th (30816 ng/g) percentiles. The maximum concentrations of PAHs were found in IBA 424 (Soba–Castro Valnera–Ordunte Burgos, Cantabria and Vizcaya, north west Spain) which presented a total concentration of 30816 ng/g, almost 20 times higher than other sites, as IBA 072 (Carrizales y Sotos de Aranjuez, Toledo, central Spain) and IBA 035 (Urdaibai–Matxitxako, Vizcaya, a UNESCO World Heritage in the north east Spain) which presented ∑PAHs of 1745 and 1738 ng/g, respectively. Overall, pyrene was the most frequently detected PAH, present in 61% of the IBAs at levels from 0.81 to 2427 ng/g, followed by benzo[b]fluoranthene present in 54% of the samples ranging from 1.04 to 6462 ng/g and benzo[k]fluoranthene detected in 53% at concentrations ranging from 1.52 to 5687 ng/g. High molecular weight PAHs (HMW, four or more rings) were more prevalent than low molecular weight PAHs (LMW, two or three rings) (Table 1). HMW mainly originated from pyrogenic sources (fossil fuel, coal, or biomass combustions), while LMW have a petrogenic origin (petroleum and its refining). The highest prevalence of HMW PAHs in front of LMW is indicative of PAHs originated from pyrogenic sources as vehicular emissions or forest fires. Aichner et al. (2013) also reported a higher prevalence of HMW than LMW PAHs in German forest soils, with ∑PAHs ranging from 105 to 14889 ng/g, slightly higher than the concentrations detected in the present study.

∑Plasticizers were detected in 50% of the IBAs and were the chemical group found at the highest mean concentrations (917 ± 118 ng/g), ranging from 5.50 to 7026 ng/g (Table 1). Because of blank contribution and the consequent high MDL, the detection frequency was low. The 50th percentile was of 9 ng/g, while the 75th percentile concentration was 2658 ng/g, and the 90th percentile concentration reached 7026 ng/g, indicating a high variability in concentrations. Plasticizers had a heterogeneous distribution among IBAs (Fig. 1). The maximum concentration was found in IBA 230 (Embalse de Marmolejo–La Ropera, Jaen, central-south Spain), where the sample was collected in a path next to a WWTP discharge, and a great amount of plastic waste and wipes were observed and documented during sampling. The main plastic-related compound was NP, detected at mean concentrations of 1155 ng/g, and ranging from 1247 to 3488 ng/g. The half-life of NP in soils is relatively short, between 1.4 and 16.7 days (Rivier et al. 2019), indicating that it does not persist in soils for extended periods of time. However, its presence in 21% of the soils can be attributed to their constant release from many sources, including pesticide formulations, irrigation with reclaimed water, use of sewage sludge as fertilizer, and breakdown of ethoxylated alkylphenols (Kim et al. 2019). Among PAEs, diethyl phthalate (DEP), diisobutyl phthalate (DiBP), and bis(2-ethylhexyl) phthalate (DEHP) were detected in 8–11% of the samples at mean concentrations ranging from 100 to 181 ng/g dw, but reaching up to 4073 ng/g. PAEs are released into the environment as a result of their use as plasticizers in a significant number of industrial and consumer plastic-based products. For instance, DEHP is used as plasticizer in the synthesis of polyvinyl chloride (PVC), and DiBP is used to provide flexibility and durability to plastic, while DEP is mainly used as additive in personal care products (nail polish, shampoos soaps, dyes) and pharmaceuticals and as solvent binders. PAEs are not chemically bonded to the plastics polymer and therefore migrate into the environment during the manufacturing, usage, and disposal of plastic materials (Prasad 2021). Once PAEs are deposited in soils, they can persist for a wide range of periods depending on their chain length. DEHP half-life in soil has been reported from 4.6 to 301 days, while the shorter chain DEP has a half-life in soil from 2 to 16 days and is decomposed more rapidly (Li et al. 2023).

∑PCBs were the chemical group detected at the lowest concentrations, despite being present in 44% of the samples, with concentrations ranging from 0.02 to 67.8 ng/g, which are in the same order of magnitude as measurements reported in European background soils (0.21 to 21 ng/g) (Schuster et al. 2011) and Canadian remote mountains (0.24 to 24 ng/g) (Abdul Hussain et al. 2019). Out of 61 IBAs with PCBs, 90% presented values below 1.5 ng/g (Fig. 1). The maximum concentrations of PCBs were detected in IBA 295 (Llanos entre Cáceres y Trujillo–Aldea del Cano, Caceres, central west Spain) which is an area with a high urban and agricultural pressure (BirdLife International 2023). Urban areas are a source of PCBs in surface soils due to atmospheric deposition, especially in the case of heavier PCBs (Yadav et al. 2017). High-chlorinated PCBs (101, 138, 153, and 180) showed a higher prevalence than low-chlorinated PCBs (28, 52), a profile related to the composition of the commonly used technical PCBs mixtures (Clorphen A60 and Aroclors 1254 and 1268), containing congeners 138, 153, and 180 as the most abundant constituents (Aichner et al. 2013). In fact, PCB 153 was the most prevalent compound detected in 39% of the soils at levels from 0.08 to 18.4 ng/g.

∑OPPs were detected in 36% of soils at levels from 1.03 to 1587 ng/g, indicative of local pollution of these compounds rather than a diffuse distribution (Fig. 1). Chlorpyrifos was the most frequently detected OPPs, present in 27% of the samples at concentrations ranging from 125 to 1587 ng/g. Due to its effectiveness against fruit and vine pests, chlorpyrifos has been one of the most widely used pesticides in southern Europe, until its ban in 2020. The agricultural soils from IBA 204 (Montes de Toledo–Cabañeros, Toledo and Ciudad Real, central-south Spain) and IBA 137 (Aiguamolls de l’Emporda, Girona, north east Spain) were the most impacted. Previous studies in Spain indicated that chlorpyrifos was an ubiquitous insecticide in the environment (García et al. 2022; Rico et al. 2021). Other OPPs were malathion, detected in 12% of the samples at levels from 1.03 to 48.3 ng/g, and chlorfenvinphos detected once at 79.3 ng/g.

∑OPEs were only detected in 5% of the samples at levels from 5.84 to 851 ng/g. The low frequency of detection is explained in part due to blank contribution and the high MDL for these compounds (Table S3). TBOEP was present in four IBAs at concentrations from 42.6 to 824 ng/g, EHDPHP twice (114 to 207 ng/g) and TDCPP and TDCEP only in one sample (Table 1). The maximum concentrations were found in IBA 036 (Montes de Izki y de Vitoria, Alava, Burgos, north Spain) in a recreational area, containing TBOEP at 824 ng/g and TDCPP at 28 ng/g. It is worth to highlight that six out of the seven IBAs where OPEs were detected showed litter around the sampling points, according to visual observation when sampling. OPEs are not chemically bound to products and can be easily released from abandoned waste materials to the environment (Wang et al. 2021). OPEs in soil have been reported at high concentrations (500 to 75000 ng/g dw) in areas contaminated by plastic and e-waste (Zapata et al. 2023), and in soils from urban cities containing 24.9 to 27900 ng/g dw (Yadav et al. 2018). Considering that IBAs are natural sites and not pollution hotspots, the low detection frequency of OPEs is expected.

### Contaminant distribution and characterization of IBAs

HCPC analysis was conducted to assess the fingerprint contamination of IBAs considering spatial data around 10 km each sampling point. PCA presented a KMO measure of 0.79, indicating a good suitability of the data for the PCA analysis. Principal components 1 and 2 explained 28.9% and 13.2% of de total variance, respectively, and grouped artificial and natural IBAs. Component 3 explained a relatively low percentage of the total variance, accounting for only 7.2% of the total variability, and differentiated those IBAs with agricultural land use affected by DDT, γ-HCH, and β-HCH from those with natural surface and artificial surface, and it was relevant to distinguish agriculture as a pollution source. Agriculture occupies almost half of the total surface in Spain, and the use of pesticides is extended to many different varieties of crops (Manjarres-López et al. 2021) which is an important factor affecting the quality of nearby IBAs. Due to its limited contribution, component 3 was not included in the clustering analysis but the results are indicated in Fig. S2.

**Fig. 2:**
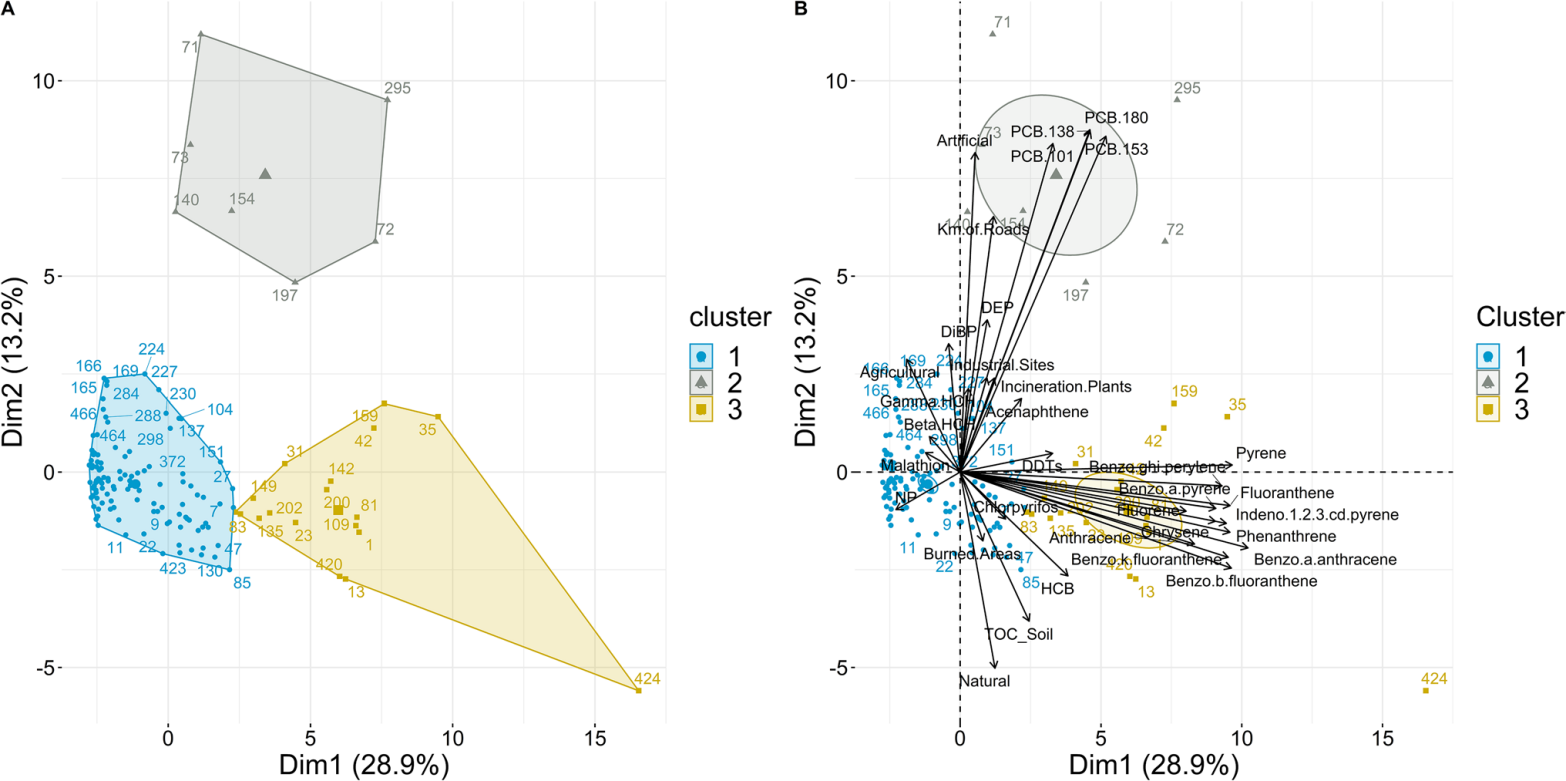
Soil HCPC analysis explaining 42.1% of the total variance, showing factor map of soil samples (**A**) and PCA (**B**). Numbers indicate IBA codes (Table S2 for IBA identification)

HCPC analysis differentiated soils samples in three clusters (Fig. 2, Table S6). Cluster 1 grouped the 115 IBAs with significantly (*p* < 0.05) lower mean concentrations of contaminants and low percentage of artificial surface and density of roads around the sampling areas. This pattern, with no clear profile of contaminants or identifiable sources, is often indicative of a diffuse pollution. Cluster 2 grouped seven IBAs with a significantly higher concentration of PCBs (101, 138, 180, 153), DEP, DiBP, pyrene, acenaphthene, and benzo[ghi]perylene. According to the land use information, the high prevalence of these compounds was also related with the presence of artificial land use, roads, industrial sites, and incineration plants. Therefore, they can be characterized as sampling points with a predominant anthropogenic pressure. This cluster includes IBAs close to large cities, as Barcelona (IBA 140) and Madrid (IBAs 71 and 73). Cluster 3 grouped IBAs with a high PAHs fingerprint and with high mean concentrations of DDTs and HCB. PAHs such as fluorene, benzo[a]anthracene, benzo[a]pyrene, indeno[1,2,3-cd]pyrene, phenanthrene, anthracene, chrysene, benzo[ghi]perylene, benzo[b]fluoranthene, pyrene, fluoranthene, and benzo[k]fluoranthene were the main contributors. As shown in Fig. 2, principal component 2 is also related to IBAs with a higher percentage of natural surface and surface affected by wildfires, which are known to be an important sources of PAHs in terrestrial ecosystems (Campos and Abrantes 2021). The maximum concentration of PAHs was found in IBA 424 (Soba–Castro Valnera–Ordunte, Burgos, Cantabria and Vizcaya, central north Spain), a sampling area that was affected by three different wildfires in less than one year before sampling. PCA also indicated that those samples also correspond to soils with a higher content of total organic carbon (TOC), which is a determinant factor in the retention of apolar compounds such as PAHs (Łyszczarz et al. 2021).

Although the PCA analysis has been useful to assess the general patterns of compounds distribution, it is important to note that the explained variance is relatively low, accounting for only 49.3% of the total variability. This indicates that there are likely other environmental variables not included in the analysis that could contribute significantly to the spatial distribution of the target compounds. For example, specific soil properties, such as texture or pH have been found to affect the retention of compounds in soil (Wenzel et al. 2002). These factors, along with other variables, may have an important influence on the results, and further investigation is needed to identify sources and understand their contribution.

### Environmental risk assessment of detected compounds

There is a lack of consensus in the model used for soil risk assessment in Europe. Different assumptions, approaches, and acceptable risk levels exist between European member states, and therefore the legal Soil Screening Values (SSVs) for organic contaminants widely differ among countries. Variations exist not only in the number of SSVs considered in each regulation, but also in their specific values (EEA 2022). The Spanish regulation sets generic reference values (GRVs) (equivalent to SSVs) of contaminants in soils with toxicity thresholds below which they do not pose a risk to human or ecosystem health (BOE 2005). However, those values only exist for 54 priority pollutants for ecosystem protection (including some legacy pesticides, PAHs, and industrial solvents), and only 10 correspond with the target compounds of our study.

Due to the lack of reference values for all compounds, we performed an ERA based on the proposed guidelines used by ECHA (European Commission 2003), defining the PNEC values following the equilibrium partitioning method approach. The RQ values for all compounds and IBAs are detailed in Table S7.

**Fig. 3:**
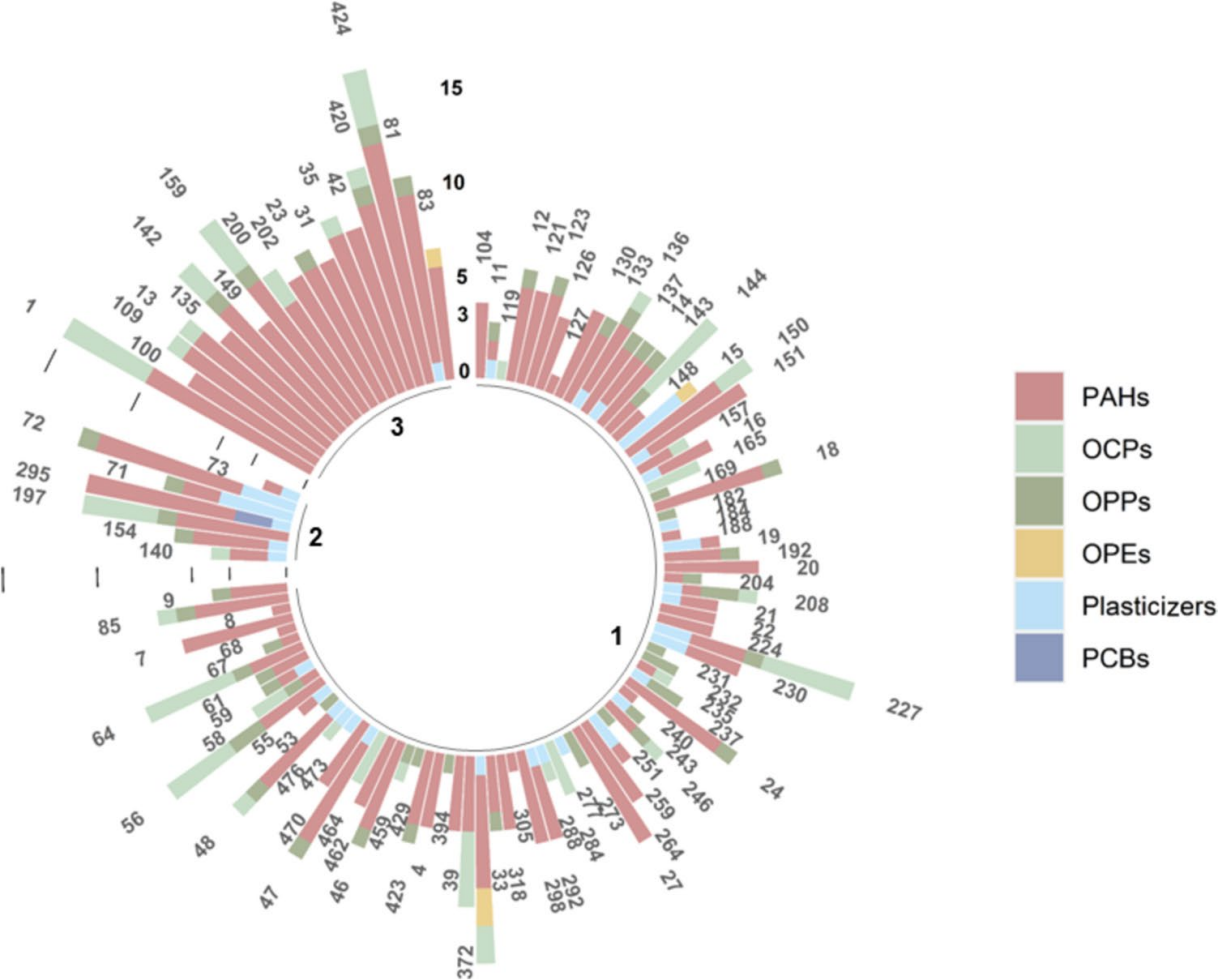
IBAs with total number of RQs > 1, classified according to identified clusters (1, 2, and 3). Numbers indicate IBA codes (Table S2 for IBA identification)

From the 140 soils samples analyzed, 95 presented at least one compound at levels above the PNEC value, indicative of concentrations of high risk (RQ > 1) for terrestrial organisms (Fig. 3). PAHs were the most frequently detected compounds exceeding the PNEC values, followed by OCPs, OPPs, plasticizers, OPEs, and PCBs. The identification of clusters among soil samples is in accordance with the risk of the chemical families identified. The majority of IBAs corresponding to cluster 1 presented a heterogeneous number of RQs values. IBAs from cluster 2 were characterized by having RQs corresponding to plasticizers, and IBA 295 (Llanos entre Cáceres y Trujillo–Aldea del Cano, Cáceres) was the only one with PCBs concentrations exceeding the PNEC values. This IBA has been included in the list of “IBAs in danger” by BirdLife International, which comprises areas where serious threats have been identified to put their natural values at risk. The main anthropogenic pressures of IBA 295 are the agricultural intensification and urbanization (BirdLife International 2023). IBAs from cluster 3 were the group with the highest number of RQs, most of them due to PAHs. RQs from OCPs and OPPs were distributed uniformly among IBAs clusters.

The RQ_f,i_ described by Zhou et al. (2019) was used to prioritize the risk compounds in IBAs soils (Table 2). Among these compounds, the OPPs chlorpyrifos and malathion were particularly concerning, as all detected residues exceeded the established PNEC values. Chlorpyrifos and malathion are among the most employed agricultural pesticides and their continued use has led to a world-wide contamination in soils (Mali et al. 2023). Both insecticides are considered highly hazardous compounds due to their neurotoxicity in non-target vertebrates (Sabzevari and Hofman 2022). The use of chlorpyrifos was banned in 2020 by the European Commission due to toxicity concerns (European Commission 2020). However, our samples were collected before this ban, although a recent report has pointed out the use of this pesticide after its derogation (Pesticide Action Network 2023). Malathion is still commercialized but only allowed in greenhouse applications (European Commission 2023b). Finally, benzo[b]fluoranthene was the cause of the highest percentage of IBAs with high-risk concentrations (54%). This is in accordance with previous studies where this PAH was frequently found exceeding the security threshold limit values in soils from protected areas in Poland (Kicińska and Dmytrowski 2023).

**Table 2:** Prioritization of concerning compounds in soil samples according to RQ_f,i_ value and percentage (%) of IBAs (*n* = 140) with environmental risk for each compound

Compound	Group	% RQ < 0.01	% 0.01 > RQ < 0.1	% 0.1 < RQ < 1	% RQ > 1	RQ_f,i_
Chlorpyrifos	OPP	72.9	0.0	0.0	27	8354
Malathion	OPP	87.9	0.0	0.0	12	681
Benzo[b]fluoranthene	PAH	46.4	0.0	0.0	54	260
Benzo[a]pyrene	PAH	82.1	0.0	0.0	18	11.2
2,4′-DDT	OCP	91.4	0.0	0.0	9.0	6.10
Benzo[ghi]perylene	PAH	55.0	0.0	0.0	45	2.91
Fluoranthene	PAH	57.9	0.0	0.0	42	1.21
Indeno[1,2,3-cd]pyrene	PAH	72.9	0.0	0.0	27	0.39
Benzo[k]fluoranthene	PAH	47.1	0.0	14.3	39	0.29
HCHs	OCP	60.7	24.3	7.1	8.0	0.15
Pyrene	PAH	39.3	1.4	33.6	26	0.10
4,4′-DDE	OCP	23.6	28.6	32.9	15	0.10
Chrysene	PAH	73.6	0.0	3.6	23	0.07
4,4′-DDD	OCP	72.1	5.7	10.7	11	0.06
DiBP	Plasticizer	88.6	0.0	0.0	11	0.02

Contamination is nowadays ubiquitous and should be taken into consideration for the conservation of habitats and biodiversity. Soil contamination can lead to its degradation and generate chain effects on biodiversity, resulting in  losses of ecosystem services such as food production. The first impact of soil contamination is observed in soil organisms. For instance, the presence of pesticides in soils has been related to alterations in the reproduction and behaviour of earthworms, nematodes, and arthropods, potentially affecting the structure and functioning of soil (Gunstone et al. 2021). The exposure to plasticizers has been linked to alterations in the reproduction and behaviour of earthworms in agricultural soils, due to their endocrine disruption, oxidative stress effects, and DNA damage (Berenstein et al. 2022; Song et al. 2019). Moreover, soil contamination is also concerning as it is an entrance of contaminants to the whole terrestrial ecosystem, as bioacumulative contaminants such as OCPs and PCBs are biomagnified along the trophic chain, reaching higher concentrations in terrestrial top predators such as mammals and birds (Cao et al. 2023; Kuo et al. 2022). Overall, contaminants present in natural areas that are hotspots of biodiversity deteriorate the ecosystems and affect the species well-being and survival, which indirectly also affects human beings. Under the current conditions of climate change, water scarcity, and human intrusion, preserving the IBAs is of utmost importance to protect and sustain innate natural richness. By establishing soil monitoring networks in IBAs, an initial assessment of the pollution status will provide useful information to implement conservation and pollution mitigation actions. These would include sustainable agriculture by minimizing the release and use of agrochemicals to IBA sites, minimizing the discharge of plastic waste, reducing and controlling the affluence of tourists, and minimizing road infrastructures and traffic. By performing regular monitoring campaigns in water (Dulsat-Masvidal et al. 2023) and soil, along with protection actions, the concentration of contaminants can be reduced in the long term. Altogether, this means reducing the anthropogenic footprint in natural areas which are biodiversity hotspots.

## Conclusions

Soils from IBAs are impacted by organic contaminants. Legacy compounds such as OCPs and PCBs were widespread in soils but present at trace levels, indicative of historic releases. In contrast, PAHs and plasticizers were found at higher concentrations, suggesting more recent pollution. OPPs and OPEs were present in specific sampling points. Three clusters with different contamination patterns were determined using PCA, and the relationships with land use were identified. Most of the IBAs were affected by diffuse pollution, as no clear sources of chemical patterns were distinguished. However, IBAs with the highest urban pressures showed the highest concentrations of PCBs and plasticizers, IBAs from north Spain had a specific PAHs contribution, and agricultural IBAs were impacted by DDT, γ-HCH, and β-HCH.

In this study, we list PNEC values based on the chemical properties and ecotoxicological information of studied contaminants in soils to evaluate the risk. A total of 95 out of 140 analyzed soils presented at least one compound at high-risk concentrations. The most concerning compounds were OPPs and PAHs, which were frequently detected at concentrations exceeding the PNEC values. Overall, the results describe for the first time the extent of soil contamination in IBAs from Spain, evidencing a widespread distribution of some contaminants in these natural areas. Further research is needed to identify the sources of contamination and propose mitigation actions to minimize the release and impact of contaminants in areas of high ecological interest.

## Supplementary Information

Below is the link to the electronic supplementary material.

Supplementary file1 (XLSX 360 kb)

## Data Availability

All data presented in this paper is available in the Supplementary Material in an excel format. It contains information on the sampling points and the concentrations of the different chemicals in the analyzed soil samples as well as predicted no-effect concentrations (PNEC).
